# Guided versus unguided internet-administered emotional awareness and expression therapy (I-EAET) for patients with persistent physical symptoms: A randomized trial

**DOI:** 10.1016/j.invent.2026.100942

**Published:** 2026-03-31

**Authors:** Daniel Maroti, Malin Ljungdahl, Helena Petersen, Mattias Rööst, Mark A. Lumley, Fredrik Falkenström, Peter Lilliengren

**Affiliations:** a Department of Psychology, Stockholm University, Stockholm, Sweden; b Department of Clinical Sciences, Lund University, Lund, Sweden; c Department of Psychology, Wayne State University, Detroit, MI, USA; d Department of Psychology, Linnaeus University, Växjö, Sweden

**Keywords:** Persistent physical symptoms, Functional somatic disorder, Internet-adminstated intervention, Emotion-focused, Psychodynamic therapy, Therapist guidance

## Abstract

Persistent physical symptoms (PPS) are common, disabling, and associated with high health care use, yet effective and scalable psychological treatments remain limited. Internet-administrated self-help programs may improve access to care. Asynchronous Internet-administered Emotional Awareness and Expression Therapy (I-EAET) with therapist guidance has been shown to reduce somatic symptoms in PPS, but the added value of the of therapist is unclear. Therefore, adults with PPS (N = 154) were randomized to guided (n = 76) or unguided (n = 78) I-EAET. Both formats comprised 10 self-help modules over 10 weeks, focusing on increasing emotional awareness, addressing unresolved conflicts, and fostering adaptive expression of avoided feelings. Guided participants received weekly written therapist feedback, whereas unguided participants had only technical support. Somatic symptom severity (PHQ-15) was assessed weekly and analyzed using linear mixed-effects models. Results showed that both groups improved significantly over time, but the group × time interaction was not significant, indicating no clear advantage of therapist guidance. However, at post-treatment, guided I-EAET was associated with a small effect size benefit in PHQ-15 scores (d = −0.21) and a higher proportion of responders (47.2% vs. 29.6%) than unguided I-EAET. Secondary outcomes (PHQ-9, GAD-7, PCL-5, DERS-16) also showed small, mostly non-significant differences favoring guidance. These small group differences were attenuated at 10-week follow-up. Taken together these findings indicate that guided and unguided I-EAET yield largely comparable outcomes for persistent physical symptoms. As the study was not powered to detect small between-group effects, these findings should be interpreted cautiously and require confirmation in adequately powered trials.

## Highlights

• The added value of therapist guidance in asynchronous internet-administered psychodynamic interventions remains unclear.
• RCT comparing guided vs unguided internet-administered I-EAET for PPS.
• No significant between-group differences on continuous somatic symptom scores although effect sizes favored guided (d = −0.21).
• A significantly higher proportion of participants achieved a clinically meaningful response in guided treatment (47% vs 30%).
• Findings inform the role of therapist guidance in internet-administered psychodynamic interventions.

## 1 Introduction

Persistent physical symptoms (PPS; Löwe et al., 2024) are somatic complaints that persist for several months or longer and are not fully attributable to structural disease or other identifiable bodily pathologies. They range from isolated symptoms and conditions such as chronic back pain or abdominal pain to complex syndromes like fibromyalgia and irritable bowel syndrome (Burton et al., 2020). PPS are linked to increased healthcare utilization (Rask et al., 2017), higher rates of unemployment and sick leave (Rask et al., 2015), reduced quality of life (Hanssen et al., 2016), and substantial psychiatric comorbidity (Henningsen et al., 2003). Without effective treatment, somatic symptoms often become chronic (Lamahewa et al., 2019).

Psychological treatment is indicated for individuals with PPS, with interventions designed to address psychological processes and mechanisms that may sustain or exacerbate symptoms and functional disability. Cognitive behavioral therapies (CBT) typically focus on modifying maladaptive thoughts and behaviors, whereas emotion-focused approaches aim to address difficulties in emotional awareness, adaptive processing, and regulation—capacities frequently impaired in patients with PPS (e.g., Schnabel et al., 2022). Although CBT is the most extensively studied approach, emotion-focused therapies have shown promising results. A recent meta-analysis found significant effects favoring emotion-focused therapies over other active treatments (including CBT) at post-treatment (*g* = −0.63) and follow-up (*g* = − 0.45) in somatic symptom disorders specifically (Lilliengren et al., 2025).

Emotional Awareness and Expression Therapy (EAET) is a short-term, emotion-focused and psychodynamically-informed intervention targeting unresolved emotional traumas and conflicts in PPS (Lumley and Schubiner, 2019a, Lumley and Schubiner, 2019b; Maroti et al., 2025a). In three randomized controlled trials (total *N* = 409), face-to-face EAET has demonstrated superior efficacy compared to CBT (Lumley et al., 2017; Yarns et al., 2020; Yarns et al., 2024). Across these three studies, about 30% of EAET participants achieved a 50% or greater reduction in pain, compared to approximately 5.5% of CBT participants.

Despite these promising results, access to EAET remains limited in many healthcare settings, particularly for patients with mobility limitations or those living in remote areas. Such barriers highlight the need for more flexible delivery formats. To address this, we developed an internet-administered version of EAET (I-EAET), delivered as a 10-module, therapist-guided self-help program (Maroti et al., 2022, Maroti et al., 2021). The intervention combines psychoeducation with experiential exercises to enhance awareness of unmet emotional needs and facilitate emotional expression, with therapist support provided through asynchronous written dialogue (see Methods section).

An uncontrolled trial of guided I-EAET with 52 participants demonstrated its feasibility and preliminary effectiveness in reducing somatic symptoms at post-treatment and at follow up at four months, with few side effects (Maroti et al., 2021). A subsequent RCT with 74 participants (Maroti et al., 2022) compared guided I-EAET to a waitlist control and found greater somatic symptom reduction at post-treatment (*d* = 0.44) and 4-month follow-up (*d* = 0.46). Approximately 20% of I-EAET participants achieved clinically significant improvement—defined as a 50% or greater reduction in symptoms—with benefits maintained at 12-month follow-up (Hallberg et al., 2025). Although these effects are modest in magnitude, they are notable given the long-standing symptom duration typical in this population, the brevity of the 10-week intervention, and the fact that the intervention was largely self-help with therapist input consisting mainly of weekly written feedback and encouragement.

While I-EAET and other internet-administered self-help programs show promise for treating various populations with PPS, the role and importance of therapist guidance in such treatments is still unclear and existing findings remains mixed. Several meta-analyses of internet-administrated psychological interventions for related conditions—such as chronic pain, fibromyalgia, and medically unexplained symptoms—have generally not identified consistent differences between guided and unguided formats in terms of symptom reduction (Buhrman et al., 2016; Bernardy et al., 2018; Van Gils et al., 2016). In contrast, a subgroup analysis of functional interference suggested that interventions without guidance produced smaller effects than those including therapist support (Vugts et al., 2018) and a more recent systematic review for chronic pain did imply that guided interventions were associated with greater clinical gains for both pain intensity and pain interference (Gandy et al., 2022).

It is important to note that “therapist guidance” is a broad term that can refer to varying degrees of therapist involvement. It can range from brief encouragement or technical reminders, to more extensive feedback on exercises, to more active therapeutic interventions. Such heterogeneity may partly explain the mixed findings regarding guidance's impact on treatment outcomes.

Further, the only randomized controlled trial comparing guided and unguided internet-administered psychodynamic therapy (IPDT)—conducted in patients with social anxiety disorder rather than PPS—found that both formats were superior to a waitlist, with larger effects for guided treatment at post-treatment but no significant differences at 6- or 12-month follow-ups (Mechler et al., 2024).

Given the mixed evidence regarding the value of therapist guidance in internet-administered interventions for patients with persistent physical symptoms (PPS)—- particularly within psychodynamic or emotion-focused approaches that emphasize the therapist as a vehicle of change (Maroti et al., 2023) - this randomized controlled trial aimed to compare guided and unguided internet-administrated Emotional Awareness and Expression Therapy (I-EAET). Emotional processing is a core element of I-EAET, but it remains unclear whether such processes require ongoing therapist input in a digital setting or whether they can be sufficiently activated through structured self-help materials alone.

Participants were randomly allocated to a 10-week intervention with or without therapist guidance. Somatic symptoms (the primary outcome) were assessed weekly during treatment and at a 10-week follow-up. Because patients with PPS tend to exhibit high levels of psychiatric comorbidity (Henningsen et al., 2003), secondary outcomes included depression, anxiety, trauma-related symptoms, and emotion regulation. Based on the only prior study comparing guided and unguided internet-administrated psychodynamic therapy (Mechler et al., 2024), we hypothesized that guided I-EAET would produce greater improvements, at least at post-treatment. The findings of this study may help clarify the role of therapist guidance in internet-administered psychodynamic treatments for PPS and inform the development of scalable intervention models.

## 2 Methods

### 2.1 Study design

**Fig. 1:**
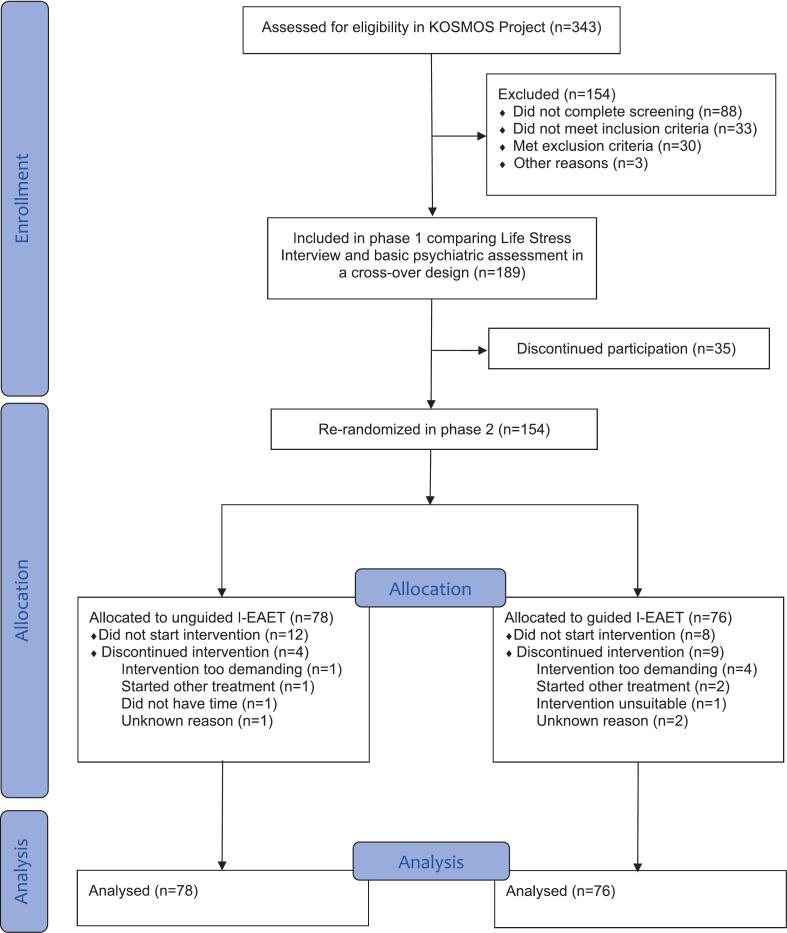
Participant flow diagram.

This study represents the second phase of the sequential longitudinal KOSMOS project, which evaluates several online/digital, emotion-focused psychological interventions for patients with persistent physical symptoms (https://www.su.se/english/research/research-projects/emotion-focused-digital-interventions-for-patients-with-medically-unexplained-symptoms). In the first phase of this project, 189 participants were randomized to receive an emotion-focused telehealth interview or a psychiatric assessment interview and then crossed conditions (Maroti et al., 2025b). All participants who did not discontinue participation in phase one moved on to the phase two in the project (see Fig. 1 for details). In the second phase, which is reported here, 154 participants where rerandomized to a two-arm randomized trial comparing guided versus unguided I-EAET. The study was approved by the Swedish Ethical Review Authority (reference number 2021-00026/03068) and pre-registered at ClinicalTrials.gov (NCT06301360). Reporting of the trial follows the CONSORT guidelines, and the completed CONSORT checklist is provided in the supplementary material.

### 2.2 Sample size

Based on the results for unguided versus guided IPDT (Mechler et al., 2024), the trial was powered to detect a between-group effect of *d* = 0.46 at post-treatment with two-tailed α = 0.05 and 80% power. Power was calculated for a mixed-effects model with 12 weekly assessments and an assumed intra-class correlation (ICC) of 0.70, consistent with prior estimates for week-to-week stability in PHQ-15 and similar symptom measures. This design reduced the required number of completers to approximately 54 per condition; allowing for 20% attrition, the adjusted target was approximately 68 per condition, or a total sample of at least 136. Our final sample of participants exceeded this number.

### 2.3 Eligibility criteria

Inclusion and exclusion criteria were defined for the main KOSMOS project. Eligible participants were adults aged 18 years or older with persistent physical symptoms lasting at least 3 months, and who had undergone a medical evaluation confirming that the symptoms were medically unexplained. Additional inclusion criteria were either a PHQ-15 score above 10 or significant burden from a specific symptom (score ≥ 2 for that symptom). Participants also had to be willing to explore emotional factors, such as stress, as possible contributors to their symptoms and to report at least one emotionally-challenging life event (defined as ≥1 on the Adverse Childhood Experience-10) or ≥21 on the PCL-5. A stable medication regimen for at least 1 month was required.

Exclusion criteria were ongoing substance abuse, severe mental illness (psychosis, pronounced suicidal ideation, or antisocial personality disorder), or current use of sedative or potentially addictive medication (e.g., benzodiazepines). Ongoing psychological treatment specifically targeting physical symptoms was an exclusion criterion; however, participants were allowed to receive other forms of usual care (e.g., pharmacological treatment or health care contacts for comorbid conditions), provided that medication was stable prior to inclusion. We did not systematically assess changes in concomitant care during the intervention phase.

### 2.4 Procedure and randomization

Recruitment for the KOSMOS Project took place between December 2023 and January 2024 through advertisements in national newspapers and on social media across Sweden, targeting individuals interested in emotion-focused digital interventions for persistent physical symptoms. Initial registration and eligibility screening were conducted via an encrypted web service (KI-BASS) requiring BankID verification. When eligibility was unclear, the study physician and psychiatrist (ML) contacted participants to clarify factors such as medication stability, suicidality, and possible substance use disorder.

In the initial project phase (March–June 2024), eligible participants were randomized to receive an emotion- and stress-focused interview (Ziadni et al., 2018) and a standard psychiatric assessment in a counterbalanced crossover design. Both interviews were delivered via videoconferencing and were associated with small but significant improvements in somatic symptoms over time (for study details, see Maroti., et al. 2025c).

Participants who remained in the project after completing the first phase were re-randomized to receive either guided or unguided I-EAET. Randomization was performed by an independent researcher unaffiliated with the study, using a computer-generated list from Random.org. The randomization list was not accessible to the authors of the study. Moreover, group assignment was revealed only after baseline assessment was completed, ensuring full allocation concealment. Participants in the guided condition were contacted by their assigned study therapist to arrange the start of I-EAET in September 2024. Participants in the unguided condition received an email that the unguided material was available to them. The treatment phase ended in November 2024, and 10-week follow-up assessments were completed in January 2025.

### 2.5 Interventions

The primary aim of I-EAET is to assist participants in overcoming difficulties related to the emotional processing of distressing feelings, often rooted in developmental trauma or stressful life events. This process aims to alleviate unregulated anxiety and defense mechanisms that can contribute to somatic symptoms. I-EAET is structured around three core treatment components: 1) psychoeducation to foster insight and awareness, 2) defense recognition and anxiety regulation through relinquishing maladaptive defenses, and 3) emotional processing and exposure. Each component is delivered via psychoeducational materials (10–15 pages per module/session) supplemented with homework assignments.

The first component, psychoeducation, comprises two modules explaining how somatic symptoms can persist in the absence of structural damage and encouraging participants to explore links between symptoms and past stressful events. The second component, defense recognition and anxiety regulation, spans two modules focused on identifying and relinquishing maladaptive defenses—particularly turning anger inward—and developing self-soothing skills through self-compassion exercises. The third component, emotional processing and exposure, includes four modules. The first two use expressive writing, adapted from intensive short-term psychodynamic therapy, to process identified trauma or stressors, while the final two focus on expressing emotions in key relationships. Homework in this phase combines written exercises with optional interpersonal tasks such as letter writing or initiating contact to address relationship issues.

The guided and unguided I-EAET programs were structured identically, consisting of 10 self-help modules delivered over a 10-week period through a secure online platform.

In the guided condition, participants received weekly written feedback from a therapist through the secure treatment platform. Feedback was provided once per week during the treatment. Participants were also able to contact their therapist during weekdays for additional support. The content of these interactions included supportive comments, encouragement to continue with the exercises, reflections on homework responses, and normalization of difficulties. There were also instances of expressive therapeutic interventions (e.g., prompting the exploration of links between emotions, anxiety, and defenses). For further examples of the range of interventions, see Maroti et al. (2023).

In the unguided condition, participants received only technical support (e.g., login or platform access issues) and no therapeutic feedback, but participants could contact a research coordinator in the event of acute crises or significant worsening of symptoms. Participants did not receive regular weekly contact.

### 2.6 Therapists

In total, 15 therapists (10 women, 5 men) provided the guided I-EAET treatment. The group included licensed psychiatrists (n = 1), licensed physicians (n = 1), and licensed psychologists (n = 4), with an average of 4.3 years (range 1–10) of post-graduation clinical experience. Additionally, nine master's-level students in the later stages of national clinical psychology programs served as study therapists. Each therapist provided guidance to an average of 4.8 participants (SD = 1.4; range 1–6).

All therapists completed an 8-h training workshop on I-EAET, led by the main developer of the program (DM). The training combined theoretical presentations with practical exercises and role-plays. Therapists were also required to read the I-EAET treatment manual and an introductory book on EAET. Weekly supervision was provided by members of the research team (ML, DM, PL) with a focus on ensuring that therapist–participant correspondence adhered to EAET principles. Although adherence was not formally monitored, supervisors regularly reviewed therapists' written feedback to participants on the online platform.

### 2.7 Primary outcome measures

Patient Health Questionnaire-15 (PHQ-15; Kroenke et al., 2010) was used as the primary outcome measure. This measure assesses the severity of 15 somatic symptoms (e.g., back pain, fatigue, constipation) experienced during the past week. Items are scored 0 (“not bothered at all”), 1 (“bothered a little”), or 2 (“bothered a lot”), yielding a total score from 0 to 30. The PHQ-15 has demonstrated good internal consistency, construct validity, acceptable test–retest reliability, and sensitivity to change (Hybelius et al., 2024). In the present study, internal consistency at project intake (n = 189) was in the acceptable range (α = 0.71).

In addition to the PHQ-15, two Visual Analogue Scales (VAS) capturing overall symptom burden and functional interference were administered, following EURONET-SOMA recommendations (Rief et al., 2017). VAS–Intensity measured the severity of bodily symptoms during the past week (“no symptoms” to “symptoms as bad as you can imagine”), and VAS–Interference measured the extent to which symptoms disrupted daily life (“no interference” to “maximum interference”). Both were presented digitally as horizontal lines, with responses recorded on a continuous 0.00–10.00 scale. Temporal reliability, estimated using intraclass correlation coefficients (ICC 2,1) from repeated assessments during the first phase of the project, was moderate for intensity (ICC = 0.57) and fair for interference (ICC = 0.51).

### 2.8 Secondary outcome measures

Patient Health Questionnaire-9 (PHQ-9; Kroenke et al., 2001) assesses depressive symptoms across nine items, each rated from 0 (“not at all”) to 3 (“nearly every day”). Total scores range from 0 to 27, with higher scores reflecting greater symptom severity. The PHQ-9 has demonstrated good reliability and validity, and in the present study internal consistency at project intake was good (α = 0.77).

Generalized Anxiety Disorder-7 item scale (GAD-7; (Spitzer et al., 2006) measures anxiety symptoms using seven items rated on the same 0–3 scale as the PHQ-9. Total scores range from 0 to 21, with higher scores indicating greater anxiety severity. Internal consistency at intake was very good (α = 0.88).

PTSD Checklist for DSM-5 (PCL-5; (Blevins et al., 2015) assesses posttraumatic stress symptoms over the past month using 20 items rated from 0 (“not at all”) to 4 (“extremely”). Total scores range from 0 to 80, with higher scores indicating more severe symptoms. The PCL-5 is psychometrically robust, and internal consistency at intake was excellent (α = 0.94).

Difficulties in Emotion Regulation Scale – 16-item version (DERS-16; Bjureberg et al., 2016) measures emotional dysregulation through 16 items rated from 1 (“almost never”) to 5 (“almost always”). Total scores range from 16 to 80, with higher scores reflecting greater difficulties in emotion regulation. The Swedish version has shown good test–retest reliability and strong convergent and discriminant validity. Internal consistency at intake was excellent (α = 0.95).

### 2.9 Data collection

The PHQ-15 was administered at pre-treatment, weekly during the 10-week intervention, at post-treatment (1 week after completion), and at a 10-week follow-up. VAS-scales and secondary measures were collected only at pre-treatment, post-treatment, and follow-up. All assessments were completed via a secure online platform on participants' preferred devices (e.g., phone, computer, tablet). Automated email and SMS reminders (up to three, 48 h apart) were sent for missed assessments, and compliance was monitored by the research team.

### 2.10 Treatment adherence

Patient adherence to treatment was measured by the average number of completed treatment modules, where a module was considered complete once the homework assignment had been submitted. In the guided condition, participants were required to submit their homework for that week in order to progress to the next module. In the unguided condition a new module was available to be opened every week.

### 2.11 Adverse events and negative effects

Adverse events were monitored continuously by the research team during the intervention period. An adverse event was defined as a) a ≥50% increase in PHQ-15 somatic symptom severity from baseline to post-treatment, or b) a participant reporting to their therapist or the research coordinator a substantial worsening of symptoms or an increase in suicidality. Any such reports were documented and, when necessary, addressed according to the study's safety protocol, which included clinical follow-up and referral to appropriate care.

Potential negative effects were assessed at the 10-week follow-up using the Negative Effect Questionnaire (NEQ; Rozental et al., 2019), a 32-item self-report instrument designed to capture a broad range of potential adverse effects of psychological treatments across emotional, cognitive, behavioral, and interpersonal domains. For each item, participants indicated whether the effect had occurred, rated its negative impact on a scale from 0 (“not at all”) to 4 (“extremely”), and specified whether they attributed the effect to the treatment. In this study, the NEQ was used descriptively to examine both the occurrence and the impact of negative effects.

### 2.12 Statistical analyses

All statistical analyses were performed using IBM SPSS Statistics, version 29.0.2.0 (IBM Corp., 2023). To evaluate the primary hypothesis, we compared the weekly rate of change in PHQ-15 scores between unguided and guided I-EAET. The data had a hierarchical structure, with repeated assessments (level 1) nested within participants (level 2), nested within treatment conditions. Linear mixed-effects models were estimated using maximum likelihood, which accommodates hierarchical data, retains all available observations (i.e., intent-to-treat [ITT]), and allows valid comparisons of fixed effects (Hesser, 2015). Time was coded 0–11 for the 12 weekly assessments, and group was coded 0 for unguided I-EAET and 1 for guided I-EAET. Models included fixed effects for time and the time × condition interaction, and random intercepts and slopes for time at the participant level.

Several covariance structures for the random effects (e.g., variance components, diagonal) were evaluated, with selection based on convergence and information criteria (smaller AIC/BIC preferred). The final model used an unstructured covariance matrix, allowing correlation between the random intercept and slope.

Missing data comprised 23.3% of the dataset, due largely to participants not starting the treatment or dropping during treatment. Maximum likelihood estimation in mixed-effects models yields unbiased estimates under the missing at random (MAR) assumption, meaning that missingness may depend on observed data but not on the unobserved outcome values themselves (Enders, 2010). MAR is generally regarded as a relatively unrestrictive and robust assumption in longitudinal clinical trial data, particularly when likely predictors of missingness are included in the model. Given that such predictors (e.g., baseline symptom severity, condition assignment, prior scores) were incorporated, and that maximum likelihood estimation naturally accommodates unbalanced data, the MAR assumption was considered reasonable. Further, although MAR cannot be formally tested, Little's MCAR test was non-significant, χ^2^(1007) = 1019.69, *p* = .385, indicating no evidence against a completely random missingness pattern.

Effect sizes (Cohen's *d*) for the PHQ-15 were derived from the fixed-effect estimates of the final mixed model without a fixed group intercept, allowing direct estimation of group means at post-treatment. Following Feingold (2009), we calculated between-group effect sizes as the estimated difference between groups at post-treatment divided by the pooled baseline standard deviation (SD = 4.65). Within-group effects were calculated as the estimated total change over the 12-week treatment period divided by the same baseline standard deviation.

Among participants with full pre–post data, clinical improvement on the PHQ-15 at post-treatment was evaluated using two definitions. First, following Hybelius et al. (2024), we applied the Minimal Clinically Important Difference (MCID), defined as a reduction of ≥3 points, reflecting change likely to be perceived as meaningful by patients with functional somatic symptoms. As a sensitivity analysis, MCID response rates were re-estimated in the intention-to-treat (ITT) sample using multiple imputation (100 imputations) to account for missing post-treatment PHQ-15 values. Second, we calculated the percentage reduction in PHQ-15 scores from pre- to post-treatment, classifying reductions of ≥30% as moderate and ≥50% as substantial responses; these thresholds are widely used in clinical research to represent meaningful symptom change. Between-group differences in response rates for each criterion were evaluated with chi-square tests.

The 10-week follow-up for the PHQ-15, VAS scales, and other secondary measures (PHQ-9, GAD-7, PCL-5, DERS-16) were analyzed using ANCOVA, with baseline scores as the covariate and group as the between-subjects factor. For the secondary outcomes, separate ANCOVAs were conducted for post-treatment and follow-up. No adjustment for family-wise error rate was applied, as these analyses were considered exploratory. Between-group effect sizes for ANCOVAs were calculated by dividing the adjusted mean difference by the pooled baseline standard deviation and interpreted as small (0.20–0.49), moderate (0.50–0.79), or large (≥0.80).

For each NEQ item, we calculated the proportion of participants endorsing a negative effect (yes/no), and if this effect where related to treatment (yes/no) and aggregated items into the standard NEQ domains (Symptoms, Quality, Hopelessness, Failure, Dependency, Stigma). Severity ratings (0–4) were used to classify effects as mild (<2), or moderate (2–3) or severe (4). Group comparisons between guided and unguided intervention were performed using independent *t*-tests (for mean number of effects) and chi-square tests (for proportions).

### 2.13 Changes and deviations from the pre-registration protocol

This study was pre-registered on ClinicalTrials.gov (NCT06301360). An oversight during data collection resulted in the Sheehan Disability Scale (SDS) being assessed only at baseline, preventing its planned use in the analyses. Although the revised Illness Perception Questionnaire (IPQ-R) was specified as an outcome measure in the trial preregistration, IPQ-R results are not reported here. Changes in illness perceptions represent a central theoretical mechanism within the broader KOSMOS trial program (phases 1–3), and a comprehensive longitudinal analysis of IPQ-R across the full trial dataset is planned in a separate publication. To avoid fragmenting the mechanistic analyses and reporting partial results from a single phase, IPQ-R outcomes are therefore not included here. Lastly, planned mediation analyses, which aimed to test whether process variables mediated the between-group treatment effect, were not conducted because, as noted below, the group × time interaction was not statistically significant, and thus the primary effect to be mediated was absent.

## 3 Results

### 3.1 Patient flow

Fig. 1 displays a CONSORT diagram of participant flow through the study. A total of 343 participants initially enrolled in the KOSMOS Project, with 154 continuing after phase 1 and re-randomized to unguided I-EAET (n = 78) or guided I-EAET (n = 76). Across both conditions, 20 (12.9%) participants never started treatment and 13 (9.7%) dropped out during the treatment phase. Following intention-to-treat principles, we analyzed data from all 154 participants.

### 3.2 Sample characteristics

The demographic and clinical characteristics of the participants are presented in Table 1. The sample was comprised mainly of middle-aged women, many of whom were married or cohabiting and well-educated. Most were employed, though a substantial proportion were on partial or full sick leave. Prior psychological treatment was almost universal, and many participants reported ongoing medication use. Participants typically described several long-standing physical symptoms combined, most often fatigue, headaches or migraine, chronic pain, and gastrointestinal complaints, with neurological symptoms also common. This pattern reflected a high overall burden and long duration of somatic distress. Psychiatric comorbidity was extensive: the majority of participants fulfilled criteria for somatic symptom disorder, often with severe presentation, while depressive disorders were highly prevalent, both current and recurrent, along with anxiety disorders. Most participants carried multiple psychiatric diagnoses, underscoring the clinical complexity of the sample. Overall, the two treatment groups were well balanced across baseline characteristics.

**Table 1:** Sample characteristics.

	Unguided I-EAET (*n* = 78)		Guided I-EAET (*n* = 76)		Total sample (*N* = 154)	
	*n* (%)	*M* (*SD*)	*n* (%)	*M* (*SD*)	*n* (%)	*M* (*SD*)
Demographics						
Age (mean, SD, and range)		45.2 (10.6) 29–72		42.1 (10.9) 22–70		43.7 (10.8) 22–72
Female	73 (93.6)		64 (84.2)		137 (89.0)	
Married and/or co-living with partner	39 (50.0)		43 (56.6)		82 (53.2)	
College or university degree	50 (64.1)		51 (67.1)		101 (65.6)	
Employed	53 (67.9)		55 (72.4)		108 (70.1)	
On sick leave (50–100%)	34 (43.6)		25 (32.9)		59 (38.3)	
Had prior psychological treatment	75 (96.2)		65 (85.5)		140 (90.9)	
Ongoing medication	57 (73.1)		51 (67.1)		108 (70.1)	

Self-reported somatic symptoms						
Fatigue	57 (73.1)		59 (77.6)		116 (75.3)	
ME/CFS	20 (25.6)		14 (18.4)		34 (22.1)	
Chronic pain	46 (59.0)		43 (56.6)		89 (57.8)	
Fibromyalgia	16 (20.5)		15 (19.7)		31 (20.1)	
Migraine or severe headache	48 (61.5)		44 (57.9)		92 (59.7)	
Whiplash	5 (6.4)		5 (6.6)		10 (6.5)	
Chest pain (non-cardiac)	9 (11.5)		15 (19.7)		24 (15.6)	
Stomach problems	47 (60.3)		40 (52.6)		87 (56.5)	
IBS	30 (38.5)		39 (51.3)		69 (44.8)	
Dyspepsia	17 (21.8)		11 (14.5)		28 (18.2)	
Throat-face-jaw	28 (35.9)		27 (35.5)		55 (35.7)	
Bruxism	19 (24.4)		15 (19.7)		34 (22.1)	
Temporomandibular dysfunction	7 (9.0)		5 (6.6)		12 (7.8)	
Tinnitus	18 (23.1)		17 (22.4)		35 (22.7)	
Neurological symptoms	35 (44.9)		36 (47.4)		71 (46.1)	
Other symptoms (e.g., shaking, muscle weakness, etc)	35 (44.9)		31 (40.8)		66 (42.9)	
Mean number of self-reported somatic symptoms		5.6 (2.8)		5.5 (3.0)		5.5 (2.9)
Mean duration of somatic symptom distress (in years)		9.5 (8.2)		8.6 (9.2)		9.1 (8.6)

Psychiatric diagnoses						
Somatic symptom disorder	61 (78.2)		57 (75.0)		118 (76.6)	
Mild	16 (20.5)		21 (27.6)			
Moderate	18 (23.1)		16 (21.1)			
Severe	27 (34.6)		20 (26.3)			
Illness anxiety disorder	7 (9.0)		6 (7.9)		13 (8.4)	
Depressive disorders						
Current	16 (20.5)		14 (18.4)		30 (19.5)	
Prior	55 (70.5)		53 (69.7)		108 (70.1)	
Recurrent	39 (50.0)		34 (44.7)		73 (47.4)	
Generalized anxiety disorder	9 (11.5)		18 (23.7)		27 (17.5)	
Social anxiety disorder	6 (7.7)		12 (15.8)		18 (11.7)	
Panic disorder	6 (7.7)		6 (7.9)		12 (7.8)	
Agoraphobia	7 (9.0)		7 (9.2)		14 (9.1)	
Obsessive compulsive disorder	6 (7.7)		4 (5.3)		10 (6.5)	
Post-traumatic stress disorder	6 (7.7)		6 (7.9)		12 (7.8)	
Eating disorder	0 (0.0)		3 (3.9)		3 (1.9)	
Mean number of psychiatric diagnoses		3.1 (1.8)		3.2 (2.0)		3.1 (1.9)

*Note.* IBS = Irritable Bowel Syndrome; ME/CFS = Myalgic Encephalomyelitis/Chronic Fatigue Syndrome.

### 3.3 Primary outcome

**Fig. 2:**
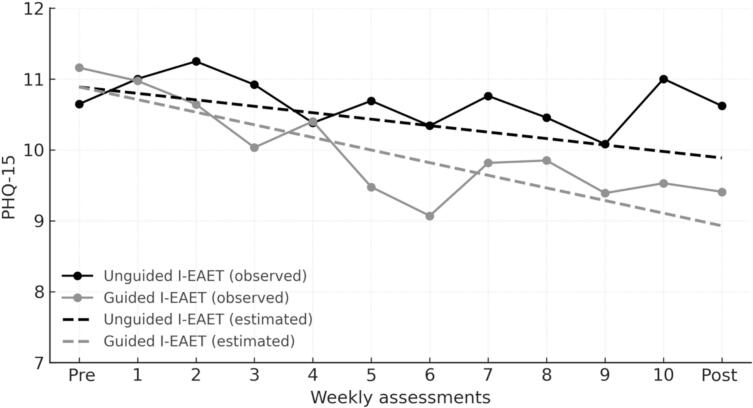
Observed and estimated PHQ-15 trajectories during treatment (*n* = 154).

Fig. 2 displays the observed and estimated PHQ-15 trajectories for unguided and guided I-EAET. The mixed-effects model showed a significant main effect of time (*b* = −0.091, SE = 0.044, 95% CI [−0.178, −0.004], *p* = .040), indicating a reduction in PHQ-15 scores over the 12-week data period in the reference group (unguided I-EAET). The group × time interaction for PHQ-15 was not statistically significant. The estimated slope difference was in the direction of greater improvement in the guided condition, but the confidence interval included zero (*b* = −0.087, SE = 0.062, 95% CI [−0.210, 0.036], *p* = .165). Over 12 weeks, unguided I-EAET showed an estimated mean reduction of −1.00 points (95% CI [−1.96, −0.04], *d* = −0.22), whereas guided I-EAET showed a reduction of −1.96 points (95% CI [−2.86, −1.06], *d* = −0.42). The adjusted between-group difference at post-treatment, estimated from the mixed-effects model, was −0.96 points (95% CI [−2.29, 0.37], *p* = .156, *d* = −0.21).

At the 10-week follow-up, ANCOVA with baseline as covariate showed that the guided group scored on average 0.39 points lower than the unguided group (95% CI [−1.46, 0.69], *p* = .480, *d* = −0.11). The complementary VAS-Intensity and VAS-Interference measures showed similar patterns, with estimated marginal means lower in the guided than unguided group, but no statistically significant between-group differences at either post-treatment or follow-up (all *p*s ≥ 0.426). Full descriptive statistics and ANCOVA estimates for primary and secondary outcomes are presented in Table 2.

**Table 2:** Observed means, standard deviations and ANCOVA-adjusted between-group differences for primary and secondary measures.

	Unguided I-EAET		Guided I-EAET				
	n	*M* (*SD*)	n	M (SD)	Adjusted mean diff^a^ [95% CI]	p	Between-group *d* [95% CI]
Primary outcomes							
PHQ-15							
Pre	76	10.6 (4.6)	70	11.2 (4.8)			
Post^b^	55	10.6 (5.8)	54	9.4 (4.9)	−0.39 [−1.46, 0.69]	0.480	−0.11 [−0.41, 0.20]
Follow-up	57	10.2 (4.6)	50	9.9 (4.8)			
VAS-intensity							
Pre	76	5.3 (1.9)	70	5.1 (2.0)			
Post	55	4.9 (2.3)	54	4.6 (2.2)	−0.11 [−0.84, 0.61]	0.757	−0.05 [−0.48, 0.32]
Follow-up	57	4.9 (1.9)	50	4.5 (2.1)	−0.22 [−0.85, 0.42]	0.496	−0.14 [−0.50, 0.25]
VAS-interference							
Pre	76	5.7 (2.5)	70	5.3 (2.4)			
Post	55	5.0 (3.0)	54	4.8 (2.9)	−0.12 [−1.00, 0.76]	0.789	−0.04 [−0.41, 0.33]
Follow-up	57	5.2 (2.6)	50	4.7 (2.8)	−0.30 [−1.06, 0.46]	0.426	−0.11 [−0.48, 0.26]

Secondary outcomes							
PHQ-9							
Pre	76	8.5 (4.8)	70	8.2 (5.2)			
Post	55	8.2 (5.0)	54	7.6 (5.7)	−0.42 [−1.95, 1.11]	0.584	−0.08 [−0.37, 0.21]
Follow-up	57	8.6 (5.1)	50	8.2 (5.5)	−0.17 [−1.89, 1.55]	0.844	−0.02 [−0.31, 0.27]
GAD-7							
Pre	76	7.1 (4.9)	70	6.7 (5.4)			
Post	55	7.6 (5.8)	54	6.5 (5.1)	−0.89 [−2.52, 0.75]	0.284	−0.14 [−0.40, 0.12]
Follow-up	57	7.6 (5.2)	49	6.4 (5.5)	−0.99 [−2.63, 0.64]	0.229	−0.23 [−0.61, 0.15]
PCL-5							
Pre	76	17.9 (14.8)	70	16.3 (17.8)			
Post	55	22.2 (13.7)	52	15.9 (17.6)	−6.16 [−11.65, −0.67]	0.028	−0.43 [−0.81, −0.05]
Follow-up	57	17.8 (17.2)	49	16.4 (19.0)	−0.46 [−5.78, 4.86]	0.865	−0.03 [−0.42, 0.37]
DERS-16							
Pre	76	40.0 (15.9)	70	39.5 (14.8)			
Post	53	36.4 (14.2)	51	35.0 (15.7)	−0.49 [−4.49, 3.51]	0.809	−0.03 [−0.42, 0.36]
Follow-up	57	38.2 (13.5)	49	36.1 (16.2)	−0.93 [−4.73, 2.87]	0.628	−0.09 [−0.48, 0.30]

Note. I-EAET = Internet-administered Emotional Awareness and Expression Therapy; PHQ-15 = Patient Heath Questionnaire 15; VAS-Intensity = Visual Analogue Scale – Intensity measure; VAS-Interference = Visual Analogue Scale – Interference measure; PHQ-9 = Patient Health Questionnaire-9; GAD-7 = Generalized Anxiety Disorder 7-item Scale; PCL-5 = PTSD Checklist for DSM-5; DERS-16 = Difficulties in Emotion Regulation Scale 16-items.

a Adjusting for baseline score.

b Results for PHQ-15 at post-treatment analyzed using mixed-effects modelling (see Results section).

### 3.4 Response rates

Among participants who completed both pre- and post-treatment assessments (n = 107), 41 individuals (38.3%) met the MCID criterion of ≥3-point reduction on the PHQ-15. Rates were 47.2% in the guided group and 29.6% in the unguided group (*p* = .048, one-sided Fisher's exact test). Sensitivity analyses based on multiple imputation in the ITT sample yielded results consistent with the completer analysis, with MCID response rates of 45% in guided I-EAET and 29% in unguided I-EAET. Using the ≥30% symptom-reduction criterion, 38 completers (35.5%) met the threshold, including 41.5% in the guided group and 29.6% in the unguided group (*p* = .140). For the ≥50% criterion, 13 completers (12.1%) reached the threshold, with rates of 17.0% in the guided group and 7.4% in the unguided group (*p* = .111). At the 10-week follow-up, the between-group differences in response rates were minimal (all *p*s > 0.350).

### 3.5 Secondary outcomes

Results are presented in Table 2. Across all secondary measures, adjusted between-group differences favored guided I-EAET over unguided I-EAET at both post-treatment and follow-up. However, all effects were negligible to small (Cohen's *d* ≤ 0.23) and non-significant (*p*s ≥ 0.229), except for post-treatment PCL-5 scores, which were significantly lower in the guided than unguided group by 6.16 points (95% CI [−11.65, −0.67], *p* = .028, *d* = −0.43, 95% CI [−0.81, −0.05]).

### 3.6 Treatment adherence

Treatment adherence by participants was high in both the guided and unguided format. Participants in the guided group submitted slightly more homework assignments (M = 7.47, SD = 2.11, *n* = 76) compared to those in the unguided group (M = 6.83, SD = 2.64, *n* = 78); however, this slight difference was not significant (independent-samples *t*-test using Welch's correction, *t*(≈150) = 1.66, *p* = .10).

Therapists sent an average of 15.9 messages (SD = 4.7) per participant, with a mean length of 244 words per message (SD = 298). Participants in the guided group either responded or initiated contact with an average of 10.5 messages (SD = 6.0) per participant, and each participant message contained an average of 154 words (SD = 181).

### 3.7 Adverse events and negative effects

Thirteen participants (8.4%) had a 50% increase in symptoms, 8 in the unguided and 5 in the guided condition, with no significant difference (*p* = .290). At follow-up, a total of 784 negative effects were reported (for further details see supplement Table 1). Of these, 591 (75.4%) were attributed to the treatment. The proportion of treatment-attributed effects did not differ significantly between the guided and unguided conditions (73.1% vs. 78.5%, *p* = .10). Among treatment-attributed effects, 348 (58.9%) were rated as at least moderate in severity (≥2), with no group differences (*p* = 1.00). Effects rated at the highest severity level were rare (23 effects; 3.9% of attributed effects) and did not significantly differ between conditions (*p* = .055). At the domain level, dependency was the most common treatment-attributed effect overall (90.1%), whereas the most moderate treatment-attributed effect was within the domain of increased symptoms (68.5%). However, highest-severity effects were uncommon (between 1.5 and 8.8%) across all domains (e.g. failure, hopelessness, stigma).

## 4 Discussion

This randomized controlled trial is the first to directly compare guided and unguided internet-administered Emotional Awareness and Expression Therapy (I-EAET) for adults with persistent physical symptoms. Both formats were associated with small but clinically meaningful reductions in somatic symptom severity in a clinically complex sample characterized by long-standing symptoms and high psychiatric comorbidity. As the trial compared two active formats of the same intervention rather than an intervention versus a control condition, large between-group differences were not expected. These findings extend previous evidence that I-EAET can reduce somatic symptom burden in this patient population.

Contrary to our hypothesis, the guided condition did not demonstrate a statistically significant advantage over the unguided format on the primary outcome or most secondary outcomes. However, some indicators favored the guided condition. In particular, a significantly larger proportion of participants in the guided group reached a minimally clinically important improvement, and effect sizes for the primary outcome were somewhat larger in the guided condition. Within-group improvements were moderate in the guided condition (PHQ-15; *d* ≈ 0.4) compared with smaller effects in the unguided condition (*d* ≈ 0.2). The magnitude of change observed in the guided condition was smaller than that reported in our previous waitlist-controlled RCT of I-EAET (*d* ≈ 1.00; Maroti et al., 2022). All participants in this study had already undergone an emotion-focused interview and psychiatric assessment online (via telehealth), which already led to some benefits (Maroti et al., 2025b). These interviews may have contributed to early symptom change and potentially attenuated differences between delivery formats. In addition to a relative larger effect size in the guided condition, a larger proportion of participants in the guided condition reached a minimally clinically important difference (≥3-point reduction on the PHQ-15; 47.2%) compared with the unguided condition (29.6%), a finding that was significant and remained robust in sensitivity analyses. These differences were modest and should be interpreted cautiously, and larger, adequately powered trials are needed to determine whether the observed differences represent a reliable effect.

Across secondary outcomes, adjusted mean differences consistently favored guided I-EAET, but effects were negligible to small and non-significant, except for a significant post-treatment advantage of guided I-EAET on PTSD symptoms. By follow-up, these differences had largely diminished. Notably, recent psychometric work has questioned the temporal and between-group measurement invariance of the PHQ-9, which may limit the interpretation of null findings for depressive symptoms (Hlynsson et al., 2025).

The lack of robust differences between guided and unguided I-EAET adds to the ongoing debate about the role of therapist guidance in internet-administrated psychological treatments. Meta-analytic work in functional somatic disorders and chronic pain has yielded inconsistent findings: while some analyses suggest stronger effects of guided interventions on interference and functioning (Vugts et al., 2018; Gandy et al., 2022), others report no systematic differences (Buhrman et al., 2016; Bernardy et al., 2018; van Gils et al., 2016). Importantly, nearly all of this research has evaluated CBT-based interventions.

The therapeutic relationship has been suggested to be central in I-EAET, where the therapist supports emotional exploration, facilitates disclosure of avoided experiences, and helps patients process emotionally meaningful conflicts (Maroti et al., 2023). From this perspective, therapist guidance might be expected to play a particularly important role in internet-administrated EAET. However, the present findings suggest a more nuanced picture. The results showed no statistically significant differences in improvement between the guided and unguided conditions, although the guided format showed somewhat larger effect sizes and a higher proportion of participants reaching a minimal clinically important change. Qualitative findings from a follow-up study of a subset of participants from this RCT further illustrate this complexity: while therapist guidance was experienced as motivating, containing, and supportive during emotionally demanding tasks, the unguided format was often described as fostering autonomy and self-reflection, but sometimes also feelings of isolation (von Below et al., 2026). These findings are consistent with the possibility that therapist involvement influences how patients engage with emotionally-demanding treatment components. It may be hypothesized that guided formats may be particularly valuable for patients who struggle to initiate or sustain emotional engagement, whereas self-guided I-EAET may be sufficient for those able to work independently with the treatment material. Such differentiation could support scalable treatment models in which therapist guidance is selectively offered to patients who need additional support.

Another important consideration concerns potential negative effects of internet-administered emotion-focused therapies. A total of 784 negative effects were reported, of which 75.4% were attributed to the treatment. Most were rated as moderate, whereas severe effects were rare (3.9%) and did not differ between formats. The most frequent treatment-attributed effects occurred within the dependency domain, which may reflect participants' perceived need for additional support during emotionally demanding treatment components. Many moderate effects were also located within the symptom domain (e.g., increased sadness or unpleasant memories), which may reflect valuable therapeutic activation of avoided affect rather than iatrogenic harm. Also, emotional activation should not be conflated with clinical deterioration. In this trial, 8.4% of participants showed ≥50% symptom worsening, with no difference between conditions. These findings highlight the importance of monitoring and safety procedures when delivering emotion-focused interventions online. Regular supervision, structured monitoring of symptom change, and access to psychiatric consultation—as implemented in the present trial—appear essential to ensuring safe delivery. Future implementation in routine care will need to replicate such supportive infrastructures to manage risk effectively.

Most previous trials of internet-administrated psychodynamic therapy (IPDT) have targeted depression and anxiety disorders (e.g., (Andersson et al., 2012; Johansson et al., 2017, Johansson et al., 2013, Johansson et al., 2012; Mechler et al., 2024; Zwerenz et al., 2017). Findings from these studies suggest that psychodynamic treatments can be successfully adapted for online formats, with effects comparable to those of internet-administrated CBT. The present study is only the second RCT to evaluate an IPDT (i.e., I-EAET) for persistent physical symptoms, thereby extending the scope of IPDT research to a population with high somatic and psychiatric comorbidity. Our findings of small but meaningful improvements in both guided and unguided formats contribute to the growing body of evidence that psychodynamic approaches can be delivered effectively online.

### 4.1 Strengths and limitations

The study has several strengths. It is the first RCT to directly compare guided and unguided formats of I-EAET, thereby addressing a critical question about the role of therapist involvement in Internet-administrated psychodynamic treatments. The sample was relatively large and clinically complex, with high levels of comorbidity and symptom burden, increasing ecological validity. The study design followed rigorous RCT methodology. Despite being relatively inexperienced, therapists were able to deliver I-EAET after receiving brief training.

At the same time, limitations must be acknowledged. Adherence—here defined at as total number of homework returned versus homework total—was somewhat lower than in previous I-EAET studies. The guided group in this study completed 75% of total homework assignments vs 85% in previous studies (Maroti et al., 2021, Maroti et al., 2022), but still comparable adherence to a study of internet-administrated CBT for somatic symptoms (Hedman-Lagerlöf and Axelsson, 2019). Moreover, the sample was predominantly female, well-educated, and experienced with psychotherapy, which may limit generalizability to more diverse populations, particularly those with lower health and digital literacy. The trial was underpowered to detect small between-group effects, as the power calculation was based on a near-medium effect size. Although efforts were made to minimize potential allegiance bias—such as having the main analyses conducted by researchers (PL, FF) independent of the treatment development and following a preregistered analytic plan—the first (DM) and fourth (MAL) authors were involved in developing the I-EAET model. While the study compared two formats of the same intervention, reducing the risk of differential allegiance effects, some interpretive bias cannot be entirely excluded. In addition, all outcomes were based on self-report measures. However, assessments were collected through standardized automated procedures, with participants completing questionnaires independently online, which reduces the risk of assessor bias. Further, we did not systematically monitor or record additional concomitant care during the intervention period. Although participants were required to have stable medication prior to inclusion and ongoing psychological treatment targeting physical symptoms was not permitted, changes in other forms of care may have occurred and could have influenced outcomes.

### 4.2 Future research

Future research could pursue several directions. First, studies should examine the cost-effectiveness of guided versus unguided formats, as the scalability of unguided interventions may come at the cost of feelings of isolation for certain patients (von Below et al., 2026). In this trial weekly supervision was provided, and the study psychiatrist was available on demand to address issues such as suicidal ideation, which a few participants reported. This highlights that the successful implementation of I-EAET may require a multidisciplinary approach, involving therapists, supervisors, and psychiatric support when necessary and these factors will of course also affect cost-effectiveness.

Second, implementation studies in routine care are needed to test I-EAET in more diverse populations, including patients with lower socioeconomic status, little or no prior psychotherapy experience, or lower health literacy. Finally, comparisons with traditional face-to-face EAET would help establish the relative benefits and limitations of internet delivery. For other treatment modalities, such as CBT, internet-administrated formats have been shown to be non-inferior to face-to-face therapy for conditions closely related to PPS (Axelsson et al., 2020) but this has still not been done for EAET.

## 5 Conclusion

In conclusion, both guided and unguided I-EAET produced modest but clinically meaningful improvements in patients with persistent physical symptoms. While therapist guidance was not associated with overall superior outcomes, it was linked to a higher proportion of clinically meaningful improvement. These findings suggest that I-EAET may be delivered effectively in both guided and self-guided formats, with therapist involvement potentially benefiting patients who require additional support in engaging with emotionally demanding treatment components. Future adequately powered trials are needed to clarify the role of therapist guidance and to inform scalable implementation strategies.

## CRediT authorship contribution statement

DM, ML, PL and MR contributed to recruitment and assessment, while HP provided logistical support. DM led the study design and initial manuscript preparation, with MAL and PL contributing to the final draft. DM, FF, PL and MAL contributed to design and methodology. All authors reviewed, commented on and approved the final manuscript.

## Ethical considerations

This study adhered to the Declaration of Helsinki, was approved by the Institutional Review Board (DNR: 2023-04956-01), and pre-registered at Clinicaltrials.gov (NCT06301360). All participants provided informed consent.

## Declaration of Generative AI and AI-assisted technologies in the writing process

During the preparation of this work the authors used ChatGPT to assist with structure and language editing. After using this tool/service, the authors reviewed and edited the content as needed and take full responsibility for the content of the published article.

## Funding statement

This study was conducted using grants received from “SU-Region Stockholm”, grant number: FoUI-979842 and “Signe and Agne Gyllenbergs stiftelse”, grant number 5631.

## Declaration of competing interest

The authors declare the following financial interests/personal relationships which may be considered as potential competing interests:

Daniel Maroti (DM) reports financial support was provided by SU-Region Stockholm and Signe and Agne Gyllenbergs stiftelse. DM declares no direct conflicts of interest, however DM has written a book on a related general topic, which could indirectly benefit from the publication of this article. M.A.L. reports personal fees from CognifiSense and fees for training health professionals in Emotional Awareness and Expression Therapy, and reports research funding outside the submitted work. Other authors declare that they have no known competing financial interests or personal relationships that could have appeared to influence the work reported in this paper.

## Data Availability

The datasets generated and analyzed during the current study are available from the corresponding author upon reasonable request.
