# Supplementary material for: Guided versus unguided internet-administered emotional awareness and expression therapy (I-EAET) for patients with persistent physical symptoms: A randomized trial
Source: Internet Interv. 2026 Mar 31;44:100942. doi: 10.1016/j.invent.2026.100942 (PMC13068840; doi:10.1016/j.invent.2026.100942)
Supplement: Supplementary file 1 — Supplementary material [file mmc1.docx]

**Supplementary Table. Negative effects at follow-up (item-level)**

**Total reported negative effects: 784**

Overall and between-group comparison

| **Outcome** | **Total n (%)** | **Guided n (%)** | **Unguided n (%)** | **p** |
| --- | --- | --- | --- | --- |
| Total effects (n) | 784 | 458 | 326 | — |
| Attributed to treatment | 591 (75.4%) | 335 (73.1%) | 256 (78.5%) | .10 |
| Moderate+ (≥2) among attributed | 348 (58.9%) | 197 (58.8%) | 151 (59.0%) | 1.00 |
| Highest severity (=4) among attributed | 23 (3.9%) | 18 (5.4%) | 5 (2.0%) | .055 |

| **NEQ Domain** | **Total effects (n)** | **Attributed n (%)** | **Moderate+ n (%)** | **Highest (=4) n (%)** |
| --- | --- | --- | --- | --- |
| Symptoms | 242 | 146 (60.3%) | 100 (68.5%) | 6 (4.1%) |
| Quality | 160 | 121 (75.6%) | 75 (62.0%) | 4 (3.3%) |
| Dependency | 101 | 91 (90.1%) | 51 (56.0%) | 8 (8.8%) |
| Stigma | 31 | 17 (54.8%) | 10 (58.8%) | 1 (5.9%) |
| Hopelessness | 82 | 68 (82.9%) | 33 (48.5%) | 1 (1.5%) |
| Failure | 111 | 93 (83.8%) | 49 (52.7%) | 2 (2.2%) |

At follow-up, a total of 784 negative effects were reported. Of these, 591 (75.4%) were attributed to the treatment. The proportion of treatment-attributed effects did not differ significantly between the guided and unguided conditions (73.1% vs. 78.5%, p = .10).

Among treatment-attributed effects, 348 (58.9%) were rated as at least moderate in severity (≥2), with no group differences (p = 1.00). Effects rated at the highest severity level were rare (23 effects; 3.9% of attributed effects) and did not significantly differ between conditions (p = .055).

At the domain level, moderate treatment-attributed effects were most frequently reported within the domain of increased symptoms. However, highest-severity effects were uncommon across all domains.
